# Methylene blue, Mycophenolic acid, Posaconazole, and Niclosamide inhibit SARS-CoV-2 Omicron variant BA.1 infection of human airway epithelial organoids

**DOI:** 10.1016/j.crmicr.2022.100158

**Published:** 2022-07-30

**Authors:** Romain Volle, Luca Murer, Anthony Petkidis, Vardan Andriasyan, Alessandro Savi, Cornelia Bircher, Nicole Meili, Lucy Fischer, Daniela Policarpo Sequeira, Daniela Katharina Mark, Alfonso Gomez-Gonzalez, Urs F. Greber

**Affiliations:** a Department of Molecular Life Sciences, University of Zürich, Winterthurerstrasse 190, 8057, Zurich, Switzerland; b Life Science Zürich Graduate School, ETH and University of Zurich, 8057 Zurich, Switzerland

**Keywords:** SARS-CoV-2 variant of concern Omicron (BA.1), Drug repurposing, Methylene blue, Mycophenolic acid, Posaconazole, Niclosamide, Persistent infection, Human nasal and bronchial epithelial explant cultures, MB, Methylene Blue, MPA, Mycophenolic acid, POS, Posaconazole, Niclo, Niclosamide, SARS-CoV-2, Severe acute respiratory syndrome coronavirus type 2, VoC, Variant of concern, HAEEC, Human airway epithelial explant culture

## Abstract

Sublineages of SARS-CoV-2 (severe acute respiratory syndrome coronavirus 2) Omicron variants continue to amass mutations in the spike (S) glycoprotein, which leads to immune evasion and rapid spread of the virus across the human population. Here we demonstrate the susceptibility of the Omicron variant BA.1 (B.1.1.529.1) to four repurposable drugs, Methylene blue (MB), Mycophenolic acid (MPA), Posaconazole (POS), and Niclosamide (Niclo) in post-exposure treatments of primary human airway cell cultures. MB, MPA, POS, and Niclo are known to block infection of human nasal and bronchial airway epithelial explant cultures (HAEEC) with the Wuhan strain, and four variants of concern (VoC), Alpha (B.1.1.7), Beta (B.1.351), Gamma (B.1.1.28), Delta (B.1.617.2) (**Weiss et al., 2021**, **Murer et al., 2022**). Our results here not only reinforce the broad anti-coronavirus effects of MB, MPA, POS and Niclo, but also demonstrate that the Omicron variant BA.1 (B.1.1.529.1) sheds infectious virus from HAEEC over at least 15 d, and maintains both intracellular and extracellular viral genomic RNA without overt toxicity, suggesting viral persistence. The data emphasize the potential of repurposable drugs against COVID-19.

## Highlights

• Methylene blue, mycophenolate, posaconazole and niclosamide inhibit SARS-CoV-2 VoC Omicron BA.1 infection of primary human airway organoids.
• Inhibition occurs upon post-exposure drug treatment and without overt cell toxicity.
• SARS-CoV-2 Omicron BA.1 infection of human airway organoids persists for at least three weeks without apparent cell lesion indicated by ORF1ab FISH staining of infected cells.

## Graphical abstract

**Figure:**
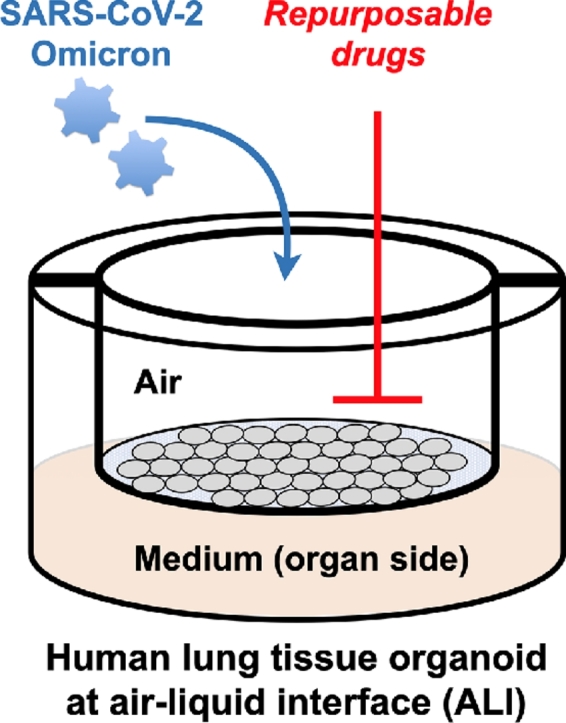

## Introduction

The causative agent of COVID-19, SARS-CoV-2 evolves in the human population at high circulation frequency despite increasing natural and vaccine-induced immunity. Variants of concern (VoC) emerge and continue to turn over. For example, the Alpha and Beta VoC were first reported in the UK and South Africa in Summer 2020, and were replaced by the Delta VoC, first reported in India October 2020. Delta soon became the dominant VoC by mid 2021 (World Health Organization, 2022, European Centre for Disease Prevention and Control (ECDC), 2022, Nextstrain, 2022). Recently, the Omicron variant emerged, as first reported in South Africa in November 2021, and became abundant worldwide from December 2021 onward, replacing Delta in the beginning of 2022 (Wei et al., 2021). Omicron variants feature up to at least 30 amino acid substitutions, several deletions and also insertions in the spike protein (S) open reading frame (ORF). Remarkably, many of the substitutions are directly localized within the receptor-binding domain (RBD) (Centers for Disease Control And Prevention, 2021), possibly the result of immune pressure upon infection and vaccination (Iketani et al., 2022, Allen et al., 2022, Rössler et al., 2022, Lauring et al., 2022). Accordingly, neutralizing antibody titers against Omicron are lower than against earlier VoC, as suggested in a preprint study from 23 laboratories (Netzl et al., 2022). Nonetheless, Omicron appears to cause less severe disease than Delta, possibly because the immune status of vaccinated or previously infected people at least partially protects against Omicron and its VoC. However, Omicron continues to cause death notably of non-vaccinated or incompletely vaccinated individuals (Lauring et al., 2022, Nyberg et al., 2022).

A growing body of evidence suggests that the multiple amino acid substitutions in the S-protein reduce the dependency of the virus on the serine protease TMPRSS2, compared to the Delta VoC (Meng et al., 2022). The altered usage of TMPRSS2 by Omicron appears to make the virion more dependent on low-pH in endosomes and the cathepsin entry pathway (Meng et al., 2022, Zhao et al., 2022, Hui et al., 2022). This highlights the considerable genetic flexibility of SARS-CoV-2 to adapt to different cell entry pathways, and renders the S-protein dependent entry a rather difficult target for powerful pan-interference strategies against SARS-CoV-2.

By engaging a multicycle drug repurposing screen against coronaviruses we previously identified and validated several broadly acting compounds against SARS-CoV-2 infection of human nasal and bronchial airway epithelial explant cultures (HAEEC) grown at air liquid interface, namely, Methylene blue (MB), Mycophenolic acid (MPA), and Posaconazole (POS) (Murer et al., 2022). These compounds have been used in the clinics for unrelated applications, and can be considered for repurposing against SARS-CoV-2. Here we report that these compounds strongly inhibit SARS-CoV-2 Omicron post exposure, along with the anti-helminthic drug niclosamide (Niclo). Finally, we provide evidence for SARS-CoV-2 persistent infection of HAEEC.

### MB, MPA and POS reduce extracellular SARS-CoV-2 Omicron progeny levels

The partial immunity against circulating Omicron subvariants BA.1 and BA.2 and continuous evolution of SARS-CoV-2 necessitate the development of an arsenal of broad acting antiviral compounds. To address this need we tested the repurposing potential of several previously identified anti-SARS-CoV-2 compounds against the Omicron variant BA.1 (B.1.1.529.1), a close relative to the currently circulating Omicron B.1.1.529 VoC, which includes BA.1, BA.2, BA.3, BA.4, BA.5 and descendent lineages (https://www.who.int/activities/tracking-SARS-CoV-2-variants). Notably, most of the amino acid changes in Omicron lineages are in the S-protein. For example, The BA.1 S-protein differs from the BA.2 one by 19 changed and five deleted amino acids, whereas BA.2 has three amino acids deleted and one inserted compared to BA.1 (Kumar et al., 2022).

We assessed the anti-Omicron potential of three compounds previously identified for their broad anti-coronavirus activity in cell culture and primary human airway epithelial cells, MB, MPA and POS (Murer et al., 2022). MB, MPA, and POS inhibit infection of primary human nasal airway epithelial explant cultures (HAEEC) by the Wuhan strain, as well as Alpha, Beta, Gamma, and Delta VoC. Although their mode-of-inhibition is not known, these compounds do not inhibit SARS-CoV-2 cell entry, and have little effects on viral genome replication, but strongly inhibit the release of infectious progeny to the apical medium, indicating that they affect one or several post replication steps, such as particle formation or egress (Murer et al., 2022).

**Fig 1:**
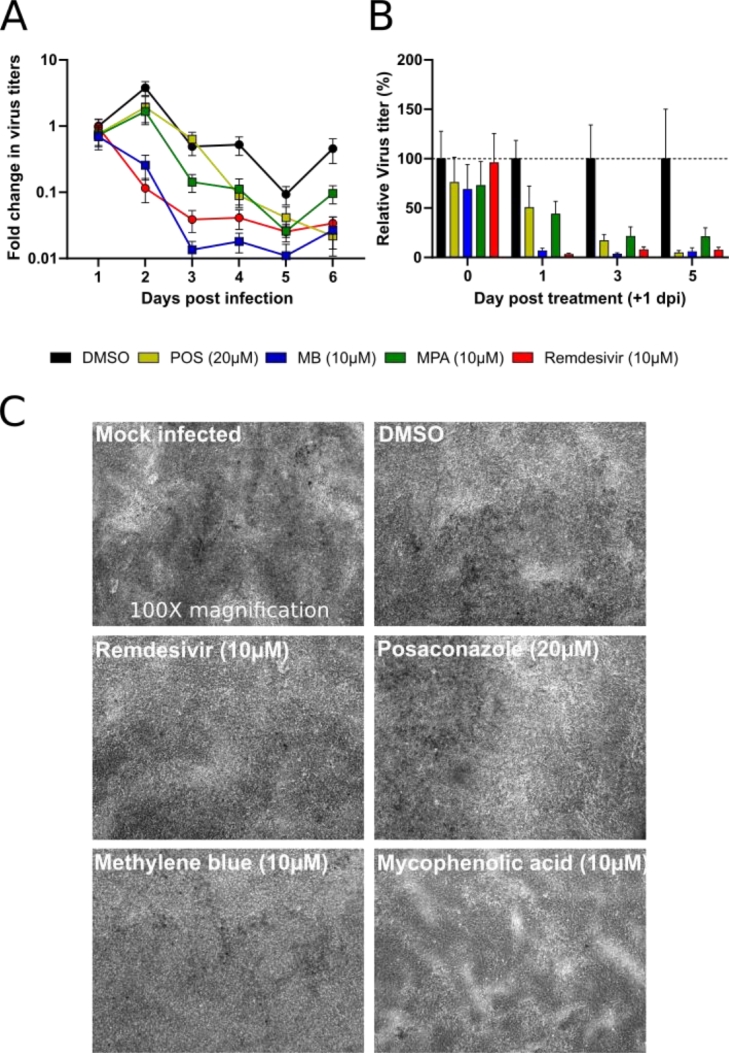
MPA, MB, and POS inhibit SARS-CoV-2 Omicron variant infection of nasal HAEEC. Antiviral effects of drug treatment represented as means ± SEM of two independent biological replicates including three and two independent technical replicates, respectively. Nasal HAEEC grown at ALI were inoculated apically with 1,000 TCID_50_ units of SARS-CoV2 Omicron variant (day 0) and subjected to drug treatments in the basolateral medium, in a post-infection regimen starting at 1d pi. MB (10 μM), MPA (10 μM), and POS (20 μM) were administered daily until 6 d. Remdesivir (10 μM) and DMSO served as drug treatment controls. SARS-CoV-2 released at the apical side was collected daily by apical washing and quantified by TCID_50_ titration. A) Global fold change in virus titer. The baseline levels represent the apical means ± SEM of virus titer from the DMSO control sample at one d pi (prior treatment). B) Relative change in virus titers of the treated inserts compared to the DMSO control. C) Microscopic images of infected / treated nasal HAEEC at 6 d pi. Pictures were taken through an inverted light microscope (Axiovert 135) at 100X magnification using CellF software (Olympus Soft Imaging Solutions GmbH, version 3.0).

Nasal HAEEC (Epithelix, MucilAir™) grown at air-liquid interface (ALI) were inoculated with Omicron BA.1 (1,000 TCID_50_ per tissue) from the apical side, followed by treatment with compounds in the basolateral medium one day (d) post infection (pi). Following five consecutive days of daily drug treatment and apical sampling, infectious virus titer was determined with TCID_50_ assays (50% tissue culture infectious dose). Treatments with MB, MPA, and POS strongly inhibited the release of Omicron progeny (Fig. 1A), similarly as previously described for the Alpha, Beta, Gamma, and Delta VoC (Murer et al., 2022). The basolateral medium had no detectable virus titer. The Omicron titers on the apical side, however, were in the range of 4.5 to 4.9-log_10_ TCID_50_/ml, as early as 1 d pi (Suppl Fig. 1). MB, MPA, and POS reduced the average SARS-CoV-2 Omicron titers, but not the release of viral genomes, in contrast to Remdesivir, albeit with different kinetics and efficiencies (Suppl Fig. 2). Compared to the DMSO-treated control cells, MB, MPA or POS reduced the infection to 6.8 (±2.3), 44.0 (±12.8), and 50.6 (±21.6)%, respectively, at 1 d post treatment, and to 5.8 (±3.9), 21.0 (±8.9) 4.8 (±2.2)% at 5 d post treatment, notably without apparent tissue lesion, basolateral medium leakage or cell toxicity (Fig. 1B and 1C). Importantly, there was no evidence that MB, MPA, and POS induced phospholipidosis (Suppl. Fig. 3). Phospholipidosis manifests itself by a foamy appearance of cytoplasmic membranes, likely owing to disregulated membrane signalling, sorting or transport (Shayman and Abe, 2013). It was reported that cationic amphiphilic drugs may have unspecific antiviral activity correlated with phospholipidosis, for example compounds such as chloroquine, that had been discussed for repurposing in the early days of COVID-19 (Tummino et al., 2021).

**Fig 2:**
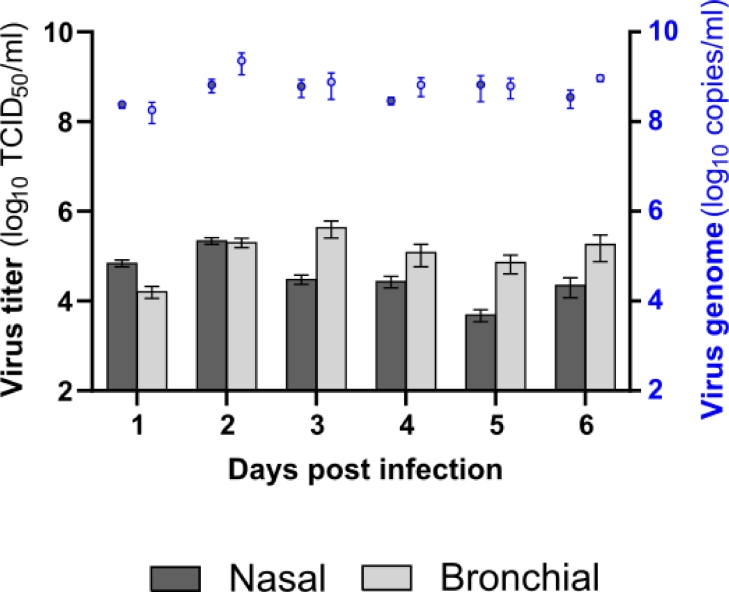
SARS-CoV-2 Omicron infection of nasal and bronchial HAEEC. Duplicate samples of nasal and bronchial HAEEC grown at ALI were inoculated apically with 1,000 TCID50 units of SARS-CoV2 Omicron. Virus released at the apical side was collected daily and quantified by TCID_50_ titration (left y-axis), and RT-qPCR using the SARS-CoV-2 M-gene (right y-axis). Data are represented as means ± SD of virus titers of five independent replicates.

**Fig 3:**
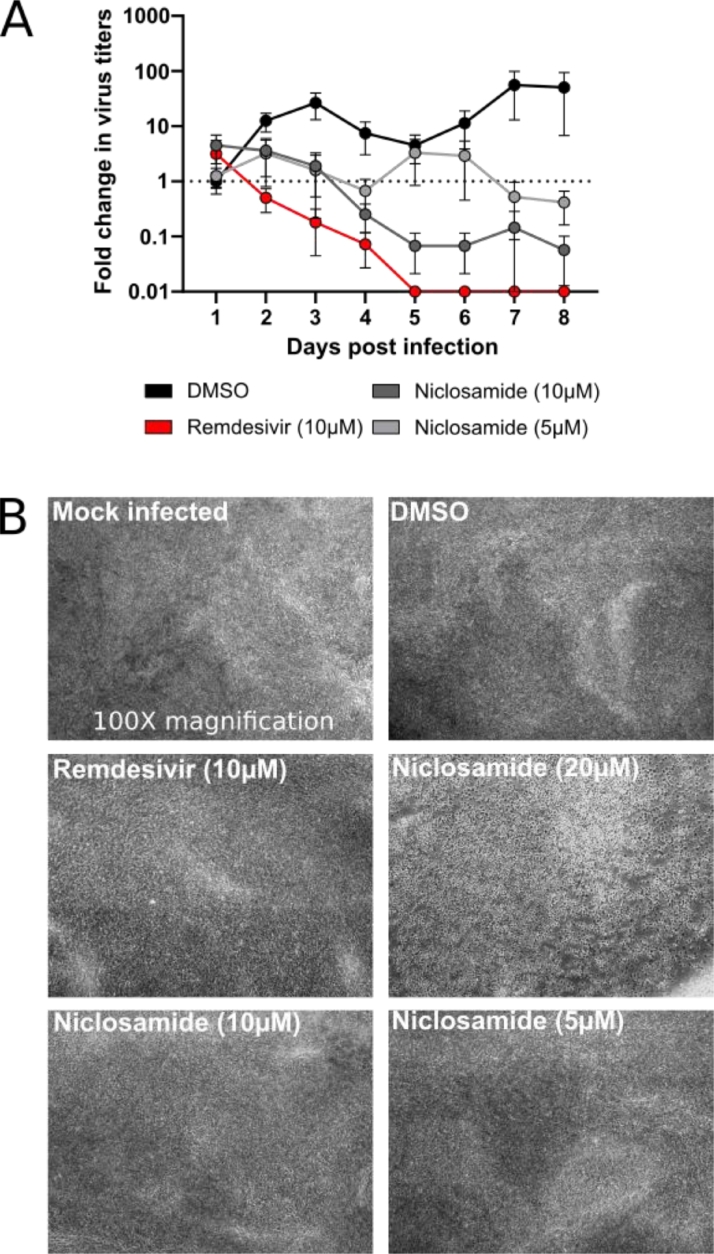
Niclo inhibits SARS-CoV-2 Omicron infection of bronchial HAEEC. Bronchial HAEEC grown at ALI were inoculated apically with 1,000 TCID_50_ units of SARS-CoV2 Omicron (0 d), and treated daily with Niclo in the basolateral medium starting at 1 d pi. Remdesivir (10 μM) and DMSO served as positive and negative controls, respectively. A) The antiviral effects of Niclo are represented as means ± SEM fold change in virus titers of two independent biological replicates and two independent technical replicates, respectively. Reference level (dotted line) represents the apical means ± SEM of virus titer in the DMSO control sample at 1 d pi. B) Microscopic images of infected / treated bronchial HAEEC at 6d pi. Pictures were taken through an inverted light microscope (Axiovert 135) at 100X magnification using CellF software (Olympus Soft Imaging Solutions GmbH, version 3.0).

### Comparable growth of SARS-CoV-2 Omicron in nasal and bronchial HAEEC

We next compared the susceptibility of human primary epithelial explant cells of nasal and bronchial origin to Omicron BA.1 infection. Our results indicate that Omicron similarly infected both nasal and bronchial lung epithelial cells (non-significant difference, multiple T-test) yielding apical titers in the range of 10^3^ to 10^6^ TCID_50_/ml (Fig. 2). These results suggest that bronchial cells can be used as a model for drug assessment against SARS-CoV-2 Omicron, in agreement with a report using *ex vivo* cultures, where Omicron exhibited higher replication in bronchial cells than in lung parenchymal cells mimicking alveoli of the lower respiratory tract (Hui et al., 2022).

### Niclosamide inhibits SARS-CoV-2 Omicron infection of bronchial HAEEC

To further address the need of acute medical treatment options with broadly available, safe and effective antivirals, we tested if Niclo inhibited Omicron infection of bronchial HAEEC. Niclo is FDA-approved for the treatment of tapeworm infections. It acts as a protonophore and has broad anti-helminthic and antiviral activity owing to its ability to neutralize acidic cytoplasmic membrane compartments, and effects on membrane trafficking and cell signaling processes (Andrews et al., 1982, Jurgeit et al., 2012, Fonseca et al., 2012, Xu et al., 2020). Recently, Niclo was shown to inhibit infection of primary human bronchial epithelial cells with SARS-CoV-2 Alpha, Beta, and Delta VoC (Weiss et al., 2021).

To test if Niclo affected SARS-CoV-2 Omicron infection, we inoculated Omicron onto bronchial HAEEC, and treated the cells with different concentrations of Niclo (20, 10, 5, and 1 µM) 1 d pi, for up to 8 d, followed by TCID_50_ titration of infectious progeny production, and virus RNA genome measurements by RT-qPCR. While 1 µM of Niclo had no effect on Omicron, and 20µM was toxic for the cells, a daily treatment with intermediate concentrations of 5 or 10 µM reduced the infectious titer of Omicron from 2-8 d by up to 2 log_10_, but not the release of viral genomes, in contrast to Remdesivir (Fig. 3 and Suppl Fig. 2). To note a concentration of 10 µM Niclo has been toxic for half of the tested inserts at d 4 of daily treatment, although we could not detect evidence that Niclo induced phospholipidosis (Suppl. Fig. 3). These results were similar to those with the Alpha, Beta, and Delta VoC reported earlier (Weiss et al., 2021). Notably, our effective concentrations of Niclo were slightly higher than those used by Weiss et al., namely 5-10 µM versus 1.25-5 µM. This difference possibly reflects the post-exposure treatment in our case, versus the preexposure treatment by Weiss and colleagues, or alternatively, donor-to-donor variability of the HAEEC. Cell-to-cell and donor-to-donor variability can have a significant influence on the infection outcome both *in vivo* and *in vitro* (Kaidashev et al., 2021, Suomalainen and Greber, 2021, Pereira et al., 2021). Taken together, Niclo may be considered as a potential inhibitor of SARS-CoV-2 with broad effects on VoC. Not surprisingly though considering the low systemic availability of Niclo (Hang et al., 2022), a recent phase 2 randomized clinical trial with per oral application of Niclo did not significantly reduce the contagious period of SARS-CoV-2 infection in a small cohort of 33 patients compared to a placebo cohort of 34 patients (Cairns et al., 2022). However, aerosolized formulations of Niclo may be worth testing against COVID-19, as they can be safely applied to human airways (Cairns et al., 2022, Backer et al., 2021).

### Persistent infection of nasal HAEEC by SARS-CoV-2

Although the origin of Omicron variant is still debated, there is increasing evidence for chronic infections. An early report of the COVID-19 Genomics Consortium UK (Rambaut et al., 2020) hypothesized that chronic infections may have played a role in the origin of the Alpha variant (B.1.1.7). This may not be far-fetched because human coronaviruses have been long known to establish and maintain persistent infections *in vitro*. For example, the alpha coronavirus CoV-229E maintains a persistent infection of human fetal lung cells (L132) for up to 300 passages over a period of two years and produces infectious progeny (Chaloner Larsson and Johnson-Lussenburg, 1981). The beta CoV-OC43 persists in infected neurons, astrocytes, microglial, and oligodendrocytes cell lines and was shown to release infectious particles for up to 25 passages and more than one hundred days (Arbour et al., 1999).

The question if SARS-CoV-2 persists *in vivo* has been debated, partly, because it is difficult to discriminate between a persistent infection and a follow-up infection. A case report from South Africa, however, provided evidence that a 22 years old, HIV-positive woman under anti-retroviral therapy was persistently infected with SARS-CoV-2 (Maponga et al., 2022). Over the course of 9 months, the virus acquired 21 nucleotide mutations, including 10 in the spike coding sequence leading to the substitution of six amino acids in the receptor binding domain (RBD), the deletion of three amino acids in the N-terminal domain, and the substitution of two amino acids in the S2 subunit. In addition, a 45 years old immunocompromised man produced infectious SARS-CoV-2 for up to 150 d, and was monitored by whole-virus genome sequencing revealing evidence for fast continuous viral evolution (Choi et al., 2020). Yet another example for long term intra-host evolution and SARS-CoV-2 persistence was reported with a diabetic male patient with Non-Hodgkin lymphoma (Bianco et al., 2022). Additionally, a study with 203 post-symptomatic patients showed evidence for SARS-CoV-2 persistence, as indicated by RT-qPCR in pharyngeal samples from 26 individuals at 15-44 d and from 5-individuals at 85-105 d post recovery (Vibholm et al., 2021).

**Fig 4:**
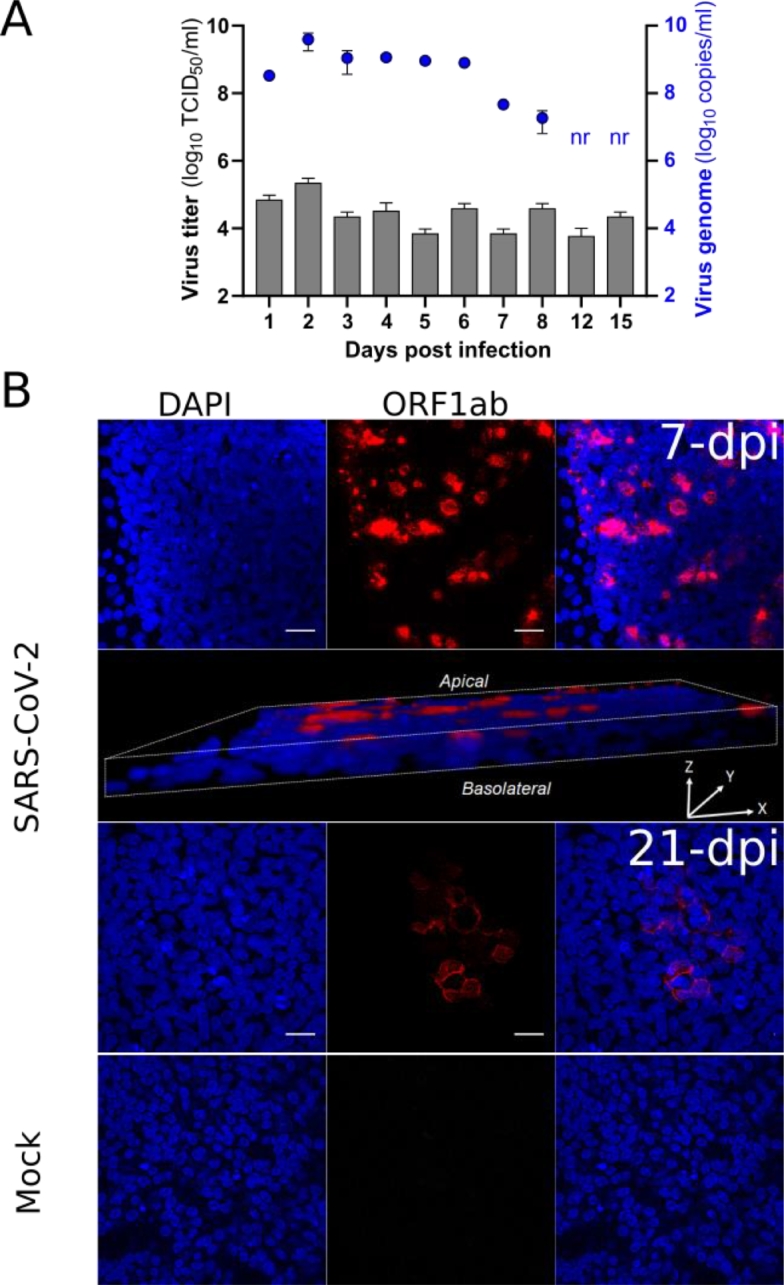
Persistent infection of nasal HAEEC by SARS-CoV-2. A) SARS-CoV-2 Omicron collected at the apical side of a duplicate (TCID_50_ means ± SD). Virus titers (TCID_50_) are shown by bars (left y-axis) and virus genome copy numbers from RT-qPCR measurements are represented by blue dots (right y-axis). The virus genome copy numbers at 12 d and 15 d pi were not tested (nr). B) Intracellular presence of SARS-CoV-2 RNA (+) genome in HAEEC fixed at 7 d and 21 d pi, respectively. SARS-CoV-2 genomes (+) were stained by RNA fluorescence in situ hybridization (FISH) using oligonucleotide probes targeting the viral ORF1ab. The scale bar represents 30 µm. A 3D projection computed by Fiji software is shown at 7 d pi. Mock infected insert fixed at 21 d pi and stained as the infected cells served as a negative control.

We took apical samples from the Omicron infected, DMSO control nasal HAEEC (presented in Fig. 1) for up to 15 d, and found a continuous titer between about 10^4^ and 10^5^ TCID50/ml in the apical milieu (Fig. 4A). The RT-qPCR genome equivalents were between about 10^7^ and 10^9^ copies / ml, indicating continued production and release of viral components over several weeks. In accordance, infected nasal HAEEC cells fixed at 7 d and 21 d pi followed by RNA FISH staining demonstrated the presence of intracellular SARS-CoV-2 ORF1ab RNA(+) fluorescent puncta predominantly in the cell layer near the apical side of the pseudo-tissue (Fig. 4B). Similarly, the 21 d infected HAEEC cells also exhibited a clear staining of the SARS-CoV-2 ORF1ab RNA(+), albeit to a lesser extent.

Together, these results support the notion that HAEEC can be infected with SARS-CoV-2 for periods of at least several weeks. This gives rise to a situation that resembles a persistent infection, where virus is steadily released without overt tissue damage. Persistence *in vivo* may enhance the chance for viral recombination, which requires that two or more different infectious agents are present in the same cell. A survey of SARS-CoV-2 genomes from UK initially observed mosaic structures of Alpha VoC and other co-circulating variants, possibly the result of recombination (Jackson et al., 2021). Other potentially recombinant SARS-CoV-2 lineage sequences were deposited in genome databases, as currently labelled XA to XL by the PANGO lineage nomenclature (Rambaut et al., 2020). As both the Delta and the Omicron VoC have been massively co-circulating in many countries across the world, it is not surprising that Delta and Omicron recombinants appear. In fact, this has recently been suggested in several independent preprints (Lacek et al., 2022, Bolze et al., 2022, Colson et al., 2022), acknowledged by the WHO with the BA.1 x AY.4 recombinant, nicknamed ‘Deltacron’, and labelled XD in the PANGO lineage nomenclature (Rambaut et al., 2020). Although it is presently unknown, if these recombinants will outcompete the known SARS-CoV-2 variants, virus recombinants raise concerns about increased transmission and pathogenicity. This issue requires continued monitoring, drug and vaccine development and explorations of repurposable drugs against COVID-19.

## Material and methods

### Viruses and chemicals

SARS-CoV-2 Omicron (B.1.1.529.1, BA.1; NH-RIVM-71076/2021) variant was obtained from the RIVM (Netherlands) through European Virus Archive global and expanded on VeroE6 cells grown in DMEM (Sigma Aldrich, Cat #D6429) supplemented with 4% of FCS (Gibco, Cat #10270-106), and 1X nonessential amino acids (NEAA, Sigma Aldrich, Cat #M7145). Virus titers were determined by TCID_50_ titration on VeroE6 cells according to the Spearman-Kärber method. Niclo was purchased to Sigma Aldrich (cat#N3510-50G; lot #BCBD3349V) and solubilized according to manufacturer's instructions. The other anti-viral compounds were purchased as described in Murer et al (Murer et al., 2022).

### Nasal and bronchial HAEEC

Human nasal HAEEC (MucilAir™, Epithelix SA, Geneva, Switzerland) cultured on transwell inserts (24-well plate) were maintained at air-liquid interface according to the supplier's instructions and cell culture medium (Epithelix SA, Geneva, Switzerland, cat#EP05MM). Nasal HAEECs were from a pool of fourteen healthy donors (Batch nr: MP010). Human bronchial cells were obtained from an individual donor (Donor 793, Epithelix SA, Geneva, Switzerland). Cells were seeded on Type IV collagen (Sigma Aldrich, cat#C5533) coated inserts (24-well plate) and expanded with PneumaCult™-Ex Basal Medium (Stemcell cat#05009) supplemented 1X with PneumaCult™-Ex 50X-Supplement (Stemcell, cat#05019) and hydrocortisone (Stemcell, cat#07925). They were differentiated with PneumaCult™-ALI Base medium (Stemcell, cat#05002) supplemented with 1X PneumaCult™-ALI (10X stock supplement, Stemcell, cat#05003), hydrocortisone (Stemcell, cat#07925), heparin (Stemcell, cat#07980), and 150 ng/mL of retinoic acid (Sigma Aldrich, cat#R2625). SARS-CoV-2 infection of nasal and bronchial HAEEC tissue, drug treatments, RNA extraction and RT-qPCR were carried out as described in Murer et al (Murer et al., 2022).

### RNA FISH with branched DNA signal amplification

Inserts were fixed with 4% PFA in PBS for 30 min at RT, washed twice with PBS, dehydrated and permeabilized with absolute methanol overnight at -20°C. Samples were rehydrated by incubation with 75%, 50%, 25%, 0% of ice-cold methanol and PBST (0.1% Tween-20 in PBS) for 5 min each. Rehydrated samples were washed on ice with a vol/vol solution of 5XSSCT / PBST for 5 min, afterwards 5XSSCT alone for 5 min. Samples were FISH-stained against SARS-CoV-2 ORF1a mRNA using ViewRNA mRNA FISH assay according to the manufacturer's instructions (ThermoFisher) with some modifications. Samples were hybridized with the SARS-CoV-2 targeting probes overnight at 40°C. The subsequent pre-amplifier, amplifier, and Alexa Fluor 546 labeled probe hybridization steps were carried out for 2 h each at 40°C. SARS-CoV-2 targeting probes custom-made #6007037-01 (ThermoFisher) were directed against the ORF1a sequences between positions 401-1327. Subsequently, cells were incubated in PBS containing DAPI for 10 min at RT. Finally, the stained inserts were detached from their plastic support,mounted on a coverslip and imaged using a SP8 confocal microscope (Leica).

#### Phospolipidosis assay

Phospolipidosis assay was performed as described by Tummino and colleagues (Tummino et al., 2021). Briefly, VeroE6 cells were seeded on a 96-well black imaging plate at a density of 15,000 cells per well and grown overnight in DMEM supplemented with 10% FCS and 1X NEAA. Then the medium was replaced with DMEM supplemented with 10% FCS, 1X NEAA, 7.5 µM NBD-PE (ThermoFisher, cat#N360), and three concentrations of MB, MPA, Niclo, NH4Cl, and Amiodarone and incubated for 24 h at 37°C. Water and DMSO were used as solvent controls. Finally, cells were stained for 20 min at 37°C with a solution of DMEM supplemented with 100 mM sodium pyruvate, 200 mM L-Glutamine, 10% FCS, 1X NEAA, 10 µg/mL Hoechst, and 2 µM Ethidium homodimer-2 (EthD-2), and imaged by onfocal microscopy (ImageXpress Micro; Molecular Devices).

## CRediT authorship contribution statement

**Romain Volle:** Supervision, Methodology, Data curation, Writing – original draft, Writing – review & editing. **Luca Murer:** Investigation, Resources. **Anthony Petkidis:** Formal analysis, Visualization, Investigation. **Vardan Andriasyan:** Data curation, Formal analysis. **Alessandro Savi:** Data curation, Investigation. **Cornelia Bircher:** Data curation, Investigation. **Nicole Meili:** Resources, Investigation. **Lucy Fischer:** Data curation, Investigation. **Daniela Policarpo Sequeira:** Data curation, Investigation. **Daniela Katharina Mark:** Data curation, Investigation. **Alfonso Gomez-Gonzalez:** Supervision, Resources, Investigation. **Urs F. Greber:** Supervision, Funding acquisition, Conceptualization, Writing – review & editing.

## Declaration of Competing Interest

The authors declare the following financial interests/personal relationships which may be considered as potential competing interests: UFG has been a consultant and stock owner in 3V-Biosciences (now Sagimet Biosciences), a consultant to F. Hoffmann-La Roche Ltd and to Union Therapeutics A/S. The funders had no role in the design of the study; in the collection, analyses, or interpretation of data; in the writing of the manuscript, or in the decision to publish the results.

## Appendix Supplementary materials

Image, application 1

Image, application 2

## References

[bib0001] Allen, H., Tessier, E., Turner, C., Anderson, C., Blomquist, P., Simons, D., et al., 2022. Comparative transmission of SARS-CoV-2 Omicron (B.1.1.529) and Delta (B.1.617.2) variants and the impact of vaccination: national cohort study, England. medRxiv. 10.1101/2022.02.15.22271001. 10.1017/S0950268823000420 PMC10125873 36938806

[bib0002] Andrews P., Thyssen J., Lorke D. (1982). The biology and toxicology of molluscicides. Bayluscide. Pharmacol. Therapeutics..

[bib0003] Arbour N., Côté G., Lachance C., Tardieu M., Cashman N.R., Talbot P.J. (1999). Acute and persistent infection of human neural cell lines by human coronavirus OC43. J. Virol..

[bib0004] Backer V., Sjöbring U., Sonne J., Weiss A., Hostrup M., Johansen H.K. (2021). A randomized, double-blind, placebo-controlled phase 1 trial of inhaled and intranasal niclosamide: A broad spectrum antiviral candidate for treatment of COVID-19. The Lancet Regional Health. Europe.

[bib0005] Bianco A., Capozzi L., Del Sambro L., Simone D., Pace L., Rondinone V. (2022). Persistent SARS-CoV-2 infection in a patient with non-Hodgkin lymphoma: intra-host genomic diversity analysis. Front. Virol..

[bib0006] Bolze, A., White, S., Basler, T., Dei Rossi, A., Roychoudhury, P., Alexander L. et al., 2022. Evidence for SARS-CoV-2 delta and omicron co-infections and recombination. medRxiv. 10.1101/2022.03.09.22272113. PMC9581791 36332633

[bib0007] Cairns D.M., Dulko D., Griffiths J.K., Golan Y., Cohen T., Trinquart L. (2022). Efficacy of Niclosamide vs Placebo in SARS-CoV-2 respiratory viral clearance, viral shedding, and duration of symptoms among patients with mild to moderate COVID-19: a phase 2 randomized clinical trial. JAMA Network Open.

[bib0008] Centers for Disease Control And Prevention. Science Brief: Omicron (B.1.1.529) Variant. 2021, access 23 March 2022; https://www.cdc.gov/coronavirus/2019-ncov/science/science-briefs/scientific-brief-omicron-variant.html. 34932278

[bib0009] Chaloner Larsson G., Johnson-Lussenburg C.M. (1981). Establishment and maintenance of a persistent infection of L132 cells by human coronavirus strain 229E. Archives of Virol..

[bib0010] Choi B., Choudhary M.C., Regan J., Sparks J.A., Padera R.F., Qiu X. (2020). Persistence and evolution of SARS-CoV-2 in an immunocompromised host. N. Engl. J. Med..

[bib0011] European Centre for Disease Prevention and Control (ECDC) SARS-CoV-2 variants dashboard, access 23 March 2022; https://www.ecdc.europa.eu/en/covid-19/situation-updates/variants-dashboard.

[bib0028] Colson P., Fournier P.E., Delerce J., Million M., Bedotto M., Houhamdi L., Yahi N., Bayette J., Levasseur A., Fantini J., Raoult D., La Scola B. (2022). Culture and identification of a “Deltamicron” SARS-CoV-2 in a three cases cluster in southern France. J. Med. Virol..

[bib0012] Fonseca B.D., Diering G.H., Bidinosti M.A., Dalal K., Alain T., Balgi A.D. (2012). Structure-activity analysis of niclosamide reveals potential role for cytoplasmic pH in control of mammalian target of rapamycin complex 1 (mTORC1) signaling. J. Biol. Chem..

[bib0013] Hang J., Chen H., Tian P., Yu R., Wang M., Zhao M. (2022). Preparation and pharmacodynamics of niclosamide micelles. J. Drug Delivery Sci. Technol..

[bib0014] Hui K., Ho J., Cheung M.C., Ng K.C., Ching R., Lai K.L. (2022). SARS-CoV-2 Omicron variant replication in human bronchus and lung ex vivo. Nature.

[bib0015] Iketani S., Liu L., Guo Y., Liu L., Chan J.F., Huang Y. (2022). Antibody evasion properties of SARS-CoV-2 Omicron sublineages. Nature..

[bib0016] Jackson, B., Rambaut, A., Pybus, O. G., Robertson, D.L., Connor, T., Loman, N. J., et al., 2021. Recombinant SARS-CoV-2 genomes involving lineage B.1.1.7 in the UK. https://virological.org/t/recombinant-sars-cov-2-genomes-involving-lineage-b-1-1-7-in-the-uk/658.

[bib0017] Jurgeit A., McDowell R., Moese S., Meldrum E., Schwendener R., Greber U.F. (2012). Niclosamide is a proton carrier and targets acidic endosomes with broad antiviral effects. PLoS Pathogens.

[bib0018] Kaidashev I., Shlykova O., Izmailova O., Torubara O., Yushchenko Y., Tyshkovska T. (2021). Host gene variability and SARS-CoV-2 infection: a review article. Heliyon.

[bib0019] Kumar, S., Karuppanan, K., Subramaniam, G., 2022. Omicron (BA.1) and sub-variants (BA.1, BA.2 and BA.3) of SARS-CoV-2 spike infectivity and pathogenicity: a comparative sequence and structural-based computational assessment. 10.1101/2022.02.11.480029. PMC9347785 35680610

[bib0020] Lacek K.A., Rambo-martin B.L., Batra D., Zheng X.Y., Hassell N., Sakaguchi H., Peacock T., Groves N., Keller M., Wilson M.M., Sheth M., Davis M.L., Borroughs M., Gerhart J., Shepard S.S., Cook P.W., Lee J., Wentworth D.E., Barnes J.R., Kondor R., Paden C.R. (2022). SARS-CoV-2 Delta-Omicron Recombinant Viruses, United States. Emerg. Infect. Dis..

[bib0021] Lauring A.S., Tenforde M.W., Chappell J.D., Gaglani M., Ginde A.A., McNeal T. (2022). Clinical severity of, and effectiveness of mRNA vaccines against, covid-19 from omicron, delta, and alpha SARS-CoV-2 variants in the United States: prospective observational study. BMJ (Clin. Res. ed.).

[bib0022] Maponga, T. G., Jeffries, M., Tegally, H., Sutherland, A. D., Wilkinson, E., Lessels, R., et al., 2022. Persistent SARS-CoV-2 infection with accumulation of mutations in a patient with poorly controlled HIV infection. Available at SSRN: https://ssrn.com/abstract=4014499 or 10.2139/ssrn.4014499. PMC9278209 35793242

[bib0023] Meng B., Abdullahi A., Ferreira I., Goonawardane N., Saito A., Kimura I. (2022). Altered TMPRSS2 usage by SARS-CoV-2 omicron impacts infectivity and fusogenicity. Nature.

[bib0024] Murer L., Volle R., Andriasyan V., Petkidis A., Gomez-Gonzalez A., Yang L. (2022). Identification of broad anti-coronavirus chemical agents for repurposing against SARS-CoV-2 and variants of concern. Current Res. Virol. Sci..

[bib0025] Netzl, A., Tureli, S., LeGresley, E., Mühlemann, B., Wilks, S. H., Smith, D. J., 2022. Analysis of SARS-CoV-2 omicron neutralization data up to 2021-12-22. bioRxiv. 10.1101/2021.12.31.474032.

[bib0026] Nextstrain, Genomic epidemiology of SARS-CoV-2 with global subsampling, access 23 March 2022; https://nextstrain.org/ncov/gisaid/global.

[bib0027] Nyberg, T., Ferguson, N. M., Nash, S. G., Webster, H. H., Flaxman, S., Andrews, N., et al., 2022. Comparative analysis of the risks of hospitalisation and death associated with SARS-CoV-2 omicron (B.1.1.529) and delta (B.1.617.2) variants in England: a cohort study. Lancet (London, England). S0140-6736(22)00462-7. Advance online publication. 10.1016/S0140-6736(22)00462-7. PMC8926413 35305296

[bib0029] Pereira N.L., Ahmad F., Byku M., Cummins N.W., Morris A.A., Owens A. (2021). COVID-19: understanding inter-individual variability and implications for precision medicine. Mayo Clin. Proceedings.

[bib0030] Rambaut A., Holmes E.C., O'Toole Á., Hill V., McCrone J.T., Ruis C. (2020). Nat. Microbiol..

[bib0031] Rambaut, A., Loman, N., Pybus, O. G., Barclay, W., Barret, J., Carabelli, A., et al., 2020. Preliminary genomic characterisation of an emergent SARS-CoV-2 lineage in the UK defined by a novel set of spike mutations. https://virological.org/t/preliminary-genomic-characterisation-of-an-emergent-sars-cov-2-lineage-in-the-uk-defined-by-a-novel-set-of-spike-mutations/563.

[bib0032] Rössler A., Riepler L., Bante D., von Laer D., Kimpel J. (2022). SARS-CoV-2 omicron variant neutralization in serum from vaccinated and convalescent persons. N. Engl. J. Med..

[bib0033] Shayman J.A., Abe A. (2013). Drug induced phospholipidosis: an acquired lysosomal storage disorder. Biochimica et biophysica acta.

[bib0034] Suomalainen M., Greber U.F. (2021). Virus infection variability by single-cell profiling. Viruses.

[bib0035] Tummino T.A., Rezelj V.V., Fischer B., Fischer A., O'Meara M.J., Monel B. (2021). Drug-induced phospholipidosis confounds drug repurposing for SARS-CoV-2. Science.

[bib0036] Vibholm L.K., Nielsen S., Pahus M.H., Frattari G.S., Olesen R., Andersen R. (2021). SARS-CoV-2 persistence is associated with antigen-specific CD8 T-cell responses. EBioMedicine.

[bib0037] Wei C., Shan K.J., Wang W., Zhang S., Huan Q., Qian W. (2021). Evidence for a mouse origin of the SARS-CoV-2 Omicron variant. J. Genetics and Genomics.

[bib0038] Weiss A., Touret F., Baronti C., Gilles M., Hoen B., Nougairède A. (2021). Niclosamide shows strong antiviral activity in a human airway model of SARS-CoV-2 infection and a conserved potency against the Alpha (B.1.1.7), Beta (B.1.351) and Delta variant (B.1.617.2). PloS one..

[bib0039] World Health Organization, weekly epidemiological update on COVID-19 –22 March 2022; https://www.who.int/publications/m.

[bib0040] Xu J., Shi P.Y., Li H., Zhou J. (2020). Broad Spectrum Antiviral Agent Niclosamide and Its Therapeutic Potential. ACS Infect. Dis..

[bib0041] Zhao H., Lu L., Peng Z., Chen L.L., Meng X., Zhang C. (2022). SARS-CoV-2 Omicron variant shows less efficient replication and fusion activity when compared with Delta variant in TMPRSS2-expressed cells. Emerging Microbes & Infect..

