# Supplementary material for: Methylene blue, Mycophenolic acid, Posaconazole, and Niclosamide inhibit SARS-CoV-2 Omicron variant BA.1 infection of human airway epithelial organoids
Source: Curr Res Microb Sci. 2022 Jul 30;3:100158. doi: 10.1016/j.crmicr.2022.100158 (PMC9338451; doi:10.1016/j.crmicr.2022.100158)
Supplement: Supplementary file 1 [file mmc1.docx]

**Supplementary Material**

**
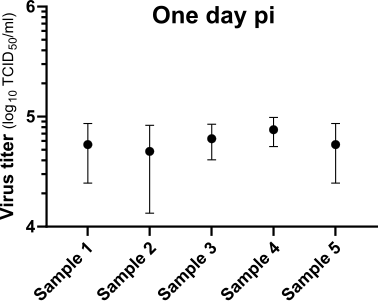
**

**Suppl Fig 1.** Apical infectious titer of SARS-CoV-2 Omicron in nasal HAEEC at 1 d pi. Data represent the means ± SD of three independent replicates of nasal HAEEC inoculated apically with 1,000 TCID_50_ units of SARS-CoV-2 Omicron.


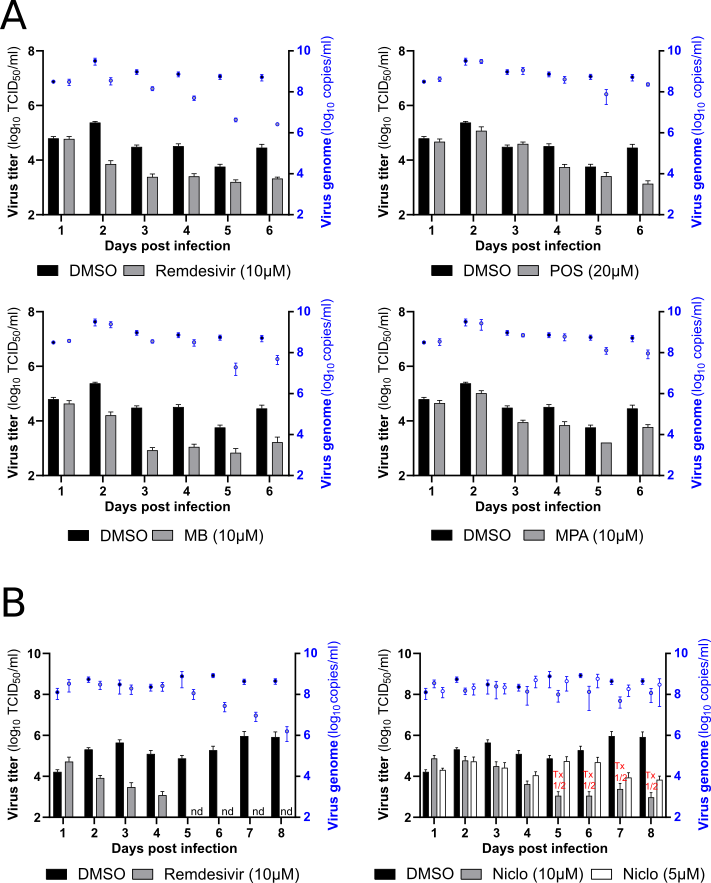


**Suppl Fig 2**. POS, MB, MPA inhibit SARS-CoV-2 infection of nasal HAEEC (A) and Niclo inhibits SARS-CoV-2 infection of bronchial HAEEC (B). Viral titer (TCID_50_/ml) readout (left y-axis) of the samples shown in Fig. 1A and 3A, and viral genome (copies/ml) readout (blue right y-axis). Data represent the mean±SD of five (A) and four independent replicates (B). Not determined (nd) indicates virus titers below 2.4 log10 TCID_50_/ml, Tx 1/2 indicates a toxicity of Nicosamide (10 µM) in 50% of the tested HAEEC ALI inserts.


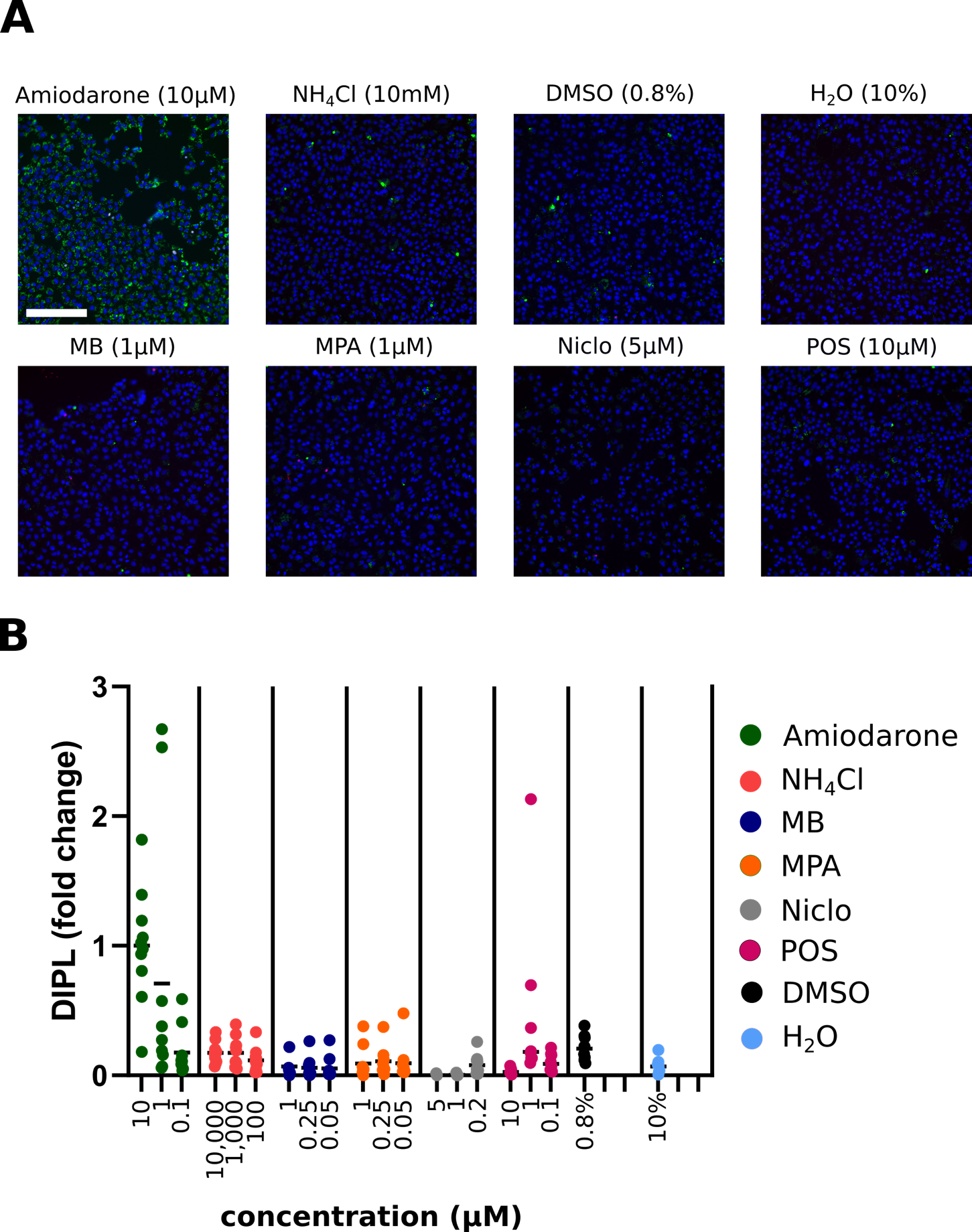


**Suppl Fig 3**. Drug-induced phospholipidosis. A) Example images of VeroE6 cells treated with the indicated drugs for 24 hours. Scale bar = 200 µm. B). Quantification of drug-induced phospholipidosis (DIPL). Fold change is calculated relative to the mean of the positive control Amiodarone.
